# Stemness-related gene signatures as a predictive tool for breast cancer radiosensitivity

**DOI:** 10.3389/fimmu.2025.1536284

**Published:** 2025-01-31

**Authors:** Jinzhi Lai, Rongfu Huang, Jingshan Huang

**Affiliations:** ^1^ Department of Oncology, The Second Affiliated Hospital of Fujian Medical University, Quanzhou, Fujian, China; ^2^ Department of Clinical Laboratory, The Second Affiliated Hospital of Fujian Medical University, Quanzhou, Fujian, China; ^3^ Department of General Surgery, The Second Affiliated Hospital of Fujian Medical University, Quanzhou, Fujian, China

**Keywords:** breast cancer, cancer stemness, radiosensitivity, PD-L1, tumor immune microenvironment

## Abstract

**Background:**

Understanding the role of cancer stemness in predicting breast cancer (BRCA) response to radiotherapy is crucial for optimizing treatment outcomes. This study developed a stemness-based signature to identify BRCA patients who are likely to benefit from radiotherapy.

**Methods:**

Gene expression data for BRCA patients were obtained from the TCGA and METABRIC databases, including 920 TCGA-BRCA and 1980 METABRIC-BRCA patients. Univariate and multivariate Cox regression analyses were used to construct a radiosensitivity signature. Immune cell infiltration and pathway enrichment analyses were conducted using ESTIMATE and GSVA methods. The TIDE algorithm and the pRRophetic platform were employed to predict responses to radiotherapy. Radioresistant BRCA cells were examined using a colony formation assay. Key genes identified in the radiosensitivity signature were validated *in vitro* by qRT-PCR.

**Results:**

By analyzing gene expression data from 920 BRCA samples, we identified a set of 267 stemness-related genes between high and low mRNAsi groups. Based on these genes, a radiosensitivity signature comprising two stemness-related genes (EMILIN1 and CYP4Z1) was constructed, stratifying patients into radiosensitive (RS) and radioresistant (RR) groups. Radiotherapy within the RS group significantly improved prognosis compared to non-radiotherapy patients. This signature was further validated in the METABRIC dataset. Notably, patients in the RS group also exhibited a significantly better response to immunotherapy compared to the RR group. We established a radioresistant BRCA cell line using the MCF-7 breast cancer cell line. A radioresistant breast cancer cell line (MCF-7/IR) was established by progressive exposure to increasing radiation doses. Comparative clonogenic and CCK8 assays demonstrated a radioresistant phenotype in the MCF-7/IR compared to MCF-7. *In vitro* studies utilizing both the MCF-7/IR and MCF-7 cell lines validated the expression of two radiosensitivity genes.

**Conclusion:**

This study identified a stemness-related gene signature predictive of radiosensitivity in breast cancer. This signature may guide personalized treatment strategies and inform the development of novel radiosensitizing agents.

## Introduction

Breast cancer (BRCA) represents the leading cause of cancer-related mortality among women worldwide, imposing a significant economic burden on healthcare systems (1). Radiotherapy has become a mainstay in breast cancer treatment, encompassing locally advanced and metastatic breast cancers (2). However, the inherent heterogeneity of breast cancer presents a significant challenge. This diversity can limit the efficacy of radiotherapy, potentially contributing to post-treatment recurrences (3). Despite these limitations, the majority of BRCA patients currently receive radiotherapy based on the type of surgery they undergo and their clinical stage, rather than on their individual radiosensitivity. Therefore, establishing novel biomarkers to improve the effectiveness of radiotherapy and overcome treatment-induced resistance holds paramount importance. This will ultimately pave the way for the development of a tailored therapeutic approach aimed at complete eradication of BRCA.

Cancer stem cells (CSCs) represent a rare subpopulation within tumors holding stemness properties characterized by their inherent ability for self-renewal (4). These cells are believed to be critical drivers of tumor initiation, progression, metastasis, recurrence, and importantly, therapeutic resistance (5). Mounting evidence suggests a pivotal role for CSCs in mediating the emergence of radio-resistance in tumors that recur after initial therapy (6, 7). CSCs exhibit enhanced mechanisms for DNA repair, autophagy, and epithelial–mesenchymal transitions (EMT), enabling them to evade radiation-induced cell death (8). In breast cancer specifically, a higher proportion of CSCs within a tumor correlates with increased resistance to ionizing radiation (9). Given this strong association, researchers are actively investigating the potential of CSC markers as clinically translatable predictors of response to radiotherapy, enabling clinicians to tailor radiotherapy regimens based on an individual tumor biology and predicted radiosensitivity.

The era of precision medicine has witnessed an unprecedented surge in the integration of genomic and transcriptomic data (10). This has revolutionized our understanding of cancer heterogeneity, leading to the identification of distinct cancer subtypes and the development of novel treatment biomarkers (11). Numerous studies have employed cellular and animal model systems to explore changes in gene and protein expression following radiation exposure, aiming to identify a robust molecular signature for predicting radiosensitivity in cancer (12–14). For instance, Torres-Roca et al. developed a 10-gene radiosensitivity index (RSI) that effectively predicted the response of 48 cancer cell lines to radiation (15). This index has undergone independent validation across diverse cancer types, including BRCA. Kim et al. established a distinct 31-gene signature derived from microarray data of NCI-60 cancer cell lines (16). This signature emerged as a significant prognostic tool for patients with BRCA undergoing radiotherapy. However, a critical knowledge gap remains regarding the potential of stemness-related signatures as biomarkers for BRCA radiotherapy response. A deeper exploration of these signatures could not only enhance our understanding of the underlying mechanisms of radio-resistance but also pave the way for selecting BRCA patients who are most likely to benefit from radiotherapy.

Our study sought to evaluate the potential of a novel stemness-related radiosensitivity signature to predict response to radiotherapy in patients with BRCA. We developed a distinct signature by integrating stemness-related genes and evaluated its effectiveness in identifying patients who would benefit from radiotherapy. By stratifying BRCA patients into radiosensitive (RS) and radioresistant (RR) groups based on this radiosensitivity risk score, we observed significant differences in therapeutic responsiveness to radiotherapy, alterations in the tumor immune microenvironment, and responses to anti-tumor therapy. These findings imply the potential of the signature to offer valuable insights into the inter-tumor heterogeneity of radiosensitivity in BRCA. Moreover, the developed radiosensitivity signature may inform the selection of optimal treatment regimens, potentially including the identification of anti-tumor drugs that exhibit synergistic effects with radiotherapy.

## Materials and methods

### Data acquisition and preprocessing

Gene expression data and associated clinical information for BRCA patients were retrieved from two publicly available databases: The UCSC Xena database (https://xena.ucsc.edu/) and The Molecular Taxonomy of Breast Cancer International Consortium (METABRIC) database (http://www.cbioportal.org/) (17, 18). The TCGA-BRCA data was downloaded in FPKM format from UCSC, normalized using log2(FPKM + 1), and converted to TPM values. In contrast, the METABRIC dataset utilized robust multi-array average (RMA) and quantile normalization methods to ensure data consistency. Inclusion criteria were established to ensure data quality and consistency: (1) Only data from primary BRCAs were included for analysis, (2) patients with complete follow-up information and a minimum follow-up duration exceeding 30 days were included, (3) availability of comprehensive radiotherapy information. Based on these criteria, a total of 920 TCGA-BRCA patients and 1980 METABRIC-BRCA patients were identified for further analysis, ensuring the presence of both RNA sequencing data and corresponding clinical information.

### Identification of stemness-related differentially expressed genes

Stemness scores (mRNAsi) for BRCA samples were obtained from a previously published study by Tathiane M. Malta et al. (19). Briefly, the authors collected gene expression data for pluripotent stem cells from the Progenitor Cell Biology Consortium database (https://www.synapse.org). This data was then employed to calculate the mRNAsi for each TCGA-BRCA tumor sample using the one-class logistic regression machine learning algorithm. The mRNAsi score is a continuous value ranging from 0 to 1, with higher scores indicating a greater degree of oncogenic dedifferentiation and enhanced stem cell-like characteristics within the tumor samples.

TCGA-BRCA patients were stratified high mRNAsi and low mRNAsi groups based on their mRNAsi scores. The median mRNAsi score served as the cut-off point for this stratification. The “limma” package was employed to identify DEGs between these two groups. The DEGs identified were further annotated as stemness-related genes (SRGs). DEGs were defined as those exceeding a false discovery rate (FDR) < 0.01 and |log2(fold change)| > 1.

### Development of the stemness-related radiosensitivity signature

A total of 920 TCGA-BRCA patients were included for signature development. Univariate Cox regression analysis was performed to identify SRGs significantly associated with overall survival (OS) in radiotherapy patients but not in non-radiotherapy patients. LASSO regression and multivariate Cox analysis were then employed to refine a radiosensitivity signature from the prognostic SRGs. signature utilizes a formula incorporating gene expression levels and corresponding coefficients to generate a radiosensitivity risk score for each patient:


$$\begin{array}{c} \text{Risk score}=(\text{Expression gene }1*\text{ Coefficient gene }1)\\ +(\text{Expression gene }2*\text{ Coefficient gene }2)+\cdot \cdot \cdot \cdot \cdot \cdot \cdot \\ +(\text{Expression gene n}*\text{ Coefficient gene n}) \end{array}$$

This risk score was then used to classify radiotherapy patients into two groups based on the median value: RS and RR. The RS group includes patients predicted to have improved OS following radiotherapy compared to those who did not receive radiotherapy. However, it is crucial to note that the predicted survival benefit of radiotherapy is not observed in the RR group for either treatment option. To validate the proportional hazards (PH) assumption of the LASSO-Cox regression analysis, we conducted the PH assumption test using the Schoenfeld residuals and the global test.

### Analysis of immune cell infiltration and functional enrichment

To evaluate the tumor microenvironment and immune cell infiltration within the RS and RR groups, the ESTIMATE algorithm was utilized to generate an estimate score (20). This score is derived from the calculation of immune score, stromal score, and ESTIMATE score. Furthermore, the CIBERSORT algorithm was employed to assess the degree of infiltration by 22 distinct immune cell types within each sample (21). Only estimates with a statistically significant p-value (p < 0.05) were considered for further evaluation. The output from CIBERSORT was then normalized, ensuring that the sum of all immune cell type fractions equals one.

To elucidate the underlying differences in cellular pathways between the two groups, we employed the Gene Set Variation Analysis (GSVA) to conduct Kyoto Encyclopedia of Genes and Genomes (KEGG) pathway enrichment analysis (22). This analysis identified the most significantly enriched molecular pathways that differed between the RS and RR groups. In addition, we further employed Gene Set Enrichment Analysis (GSEA) to gain a more comprehensive understanding of the biological processes (BP), molecular functions (MF), and cellular components (CC) associated with the RS and RR groups.

### Prediction of immunotherapy, chemotherapy and targeted-therapy response

To predict the potential response of BRCA patients to immunotherapy, we employed the Tumor Immune Dysfunction and Exclusion (TIDE) scores retrieved from the TIDE portal (http://tide.dfci.harvard.edu/) (23). The TIDE score, T cell dysfunction score and T cell exclusion score, derived from the transcriptome data of BRCA patients, represent the likelihood of response to immunotherapy. Additionally, we obtained Immunophenotype scores (IPS) specific to CTLA-4 and PD-1 blockade from The Cancer Immunome Atlas (TCIA) database(https://tcia.at/home) (24). These IPS scores, calculated using the expression levels of representative gene sets and ranging from 0 to 10, further inform the predicted efficacy of immunotherapy for each patient. For the prediction of chemotherapeutic and targeted-therapy response, we utilized the pRRophetic R package (25). This package leverages data from the Genomics of Drug Sensitivity in Cancer (GDSC) database to predict drug sensitivity based on the concept of half-maximal inhibitory concentration (IC50).

### Colony formation assay

To evaluate clonogenic survival, MCF-7 and MCF-7/IR cells were plated at a density of 100 cells per well in six-well plates and allowed to adhere overnight. The next day, the cells were exposed to escalating doses of radiation (0, 5 and 10 Gy). The cultures were then maintained for a period of 10-14 days, during which colonies became visible. After fixation with 4% paraformaldehyde for 30 minutes, the colonies were stained with 1% crystal violet solution for 30 minutes, with the dye solution being reused to minimize waste. The efficiency of colony formation was calculated as the ratio of the number of colonies to the initial number of seeded cells, expressed as a percentage.

### Cell culture and establishment of radioresistant BRCA cells

Human breast cancer cell line MCF-7 was purchase from the American Type Culture Collection (Manassas, USA, Lot Number: 70019550). Both MCF-7 and MCF-7/IR cell lines were cultured under identical conditions in Minimum Essential Medium supplemented with 10% fetal bovine serum (Corning, United States) and 1% penicillin-streptomycin antibiotics (Gibco-BRL, United States). To establish the radioresistant MCF-7/IR cell line, MCF-7 cells were subjected to a regimen of fractionated ionizing radiation. The total dose delivered was 60 Gy of γ-irradiation, administered in 2 Gy fractions, five times per week for a total of six weeks. Parental MCF-7 cells underwent a sham irradiation procedure during the same period, serving as the non-irradiated control group for subsequent experiments. The radio-resistance of MCF-7/IR cells was assessed using the CCK-8 assay. MCF-7/IR and MCF-7 cells were seeded in 96-well plates, irradiated (0, 5, or 10 Gy), and incubated with CCK-8 solution. Cell viability was proportional to the OD450 measured using a microplate reader.

### Quantitative real-time polymerase chain reaction

Total RNA was extracted from the cells using trizol reagent (Invitrogen, San Diego, CA, USA), following the manufacturer’s protocol. The concentration and purity of the extracted RNA were assessed using a NanoDrop 2000 spectrophotometer (Thermo Fisher Scientific, USA). Quantitative real-time PCR was performed to quantify the mRNA expression levels of target genes. The SYBR Prime Script RT-PCR Kit (Invitrogen, USA) was used according to the manufacturer’s instructions. The specific primer sequences for the target genes of interest are listed in **Supplementary Table S1**. All qRT-PCR experiments were conducted in triplicate, incorporating appropriate negative controls to rule out non-specific amplification. The threshold cycle (Ct) values obtained for each target gene were normalized to the geometric mean of Ct values for a panel of internal control genes, including GAPDH. Relative gene expression levels were calculated using the 2^-ΔΔCt^ method and are presented as fold change relative to the normalized internal controls.

### Flow cytometry

To assess PD-L1 protein expression, flow cytometry was employed. Twenty-four hours post-irradiation (5 Gy), MCF-7 and MCF-7/IR cells were harvested and prepared for analysis. Single-cell suspensions were stained with anti-human CD274 (PD-L1; clone MIH1) and an isotype control antibody (IgG1 kappa; clone P3.6.2.8.1), both obtained from eBioscience. Following incubation and washing, cells were analyzed using a BD FACSVerse flow cytometer (BD Biosciences, USA). Data acquisition and analysis were performed using FlowJo software version 10 (BD Biosciences, USA). Mean fluorescence intensity (MFI) was calculated for each sample to quantify PD-L1 expression levels.

### Statistical analysis

Statistical analyses were performed using R software version 4.1.3. The choice of statistical test was guided by the nature of the data being analyzed. For comparisons between categorical variables or pairwise features among different groups, the Chi-square test was employed. The Mann-Whitney U test was used to determine statistically significant differences between two groups. For comparisons between categorical variables or pairwise features among multiple independent groups, the Kruskal-Wallis test was employed. Pearson’s correlation coefficient was used to evaluate linear relationships between normally distributed variables. Alternatively, Spearman’s rank correlation coefficient was utilized for non-parametric data exhibiting non-normal distributions. To analyze differences in survival outcomes between two or more groups, Kaplan-Meier curves were generated, and the log-rank test was employed to assess statistical significance. All statistical tests were two-tailed, allowing for the detection of both positive and negative associations. A significance level of p < 0.05 was utilized throughout the study. This threshold was applied unless otherwise specified in the analysis.

## Results

### The relationship of cancer stemness with radiosensitivity and clinicopathological characteristics of TCGA-BRCA patients

**Figure 1:**
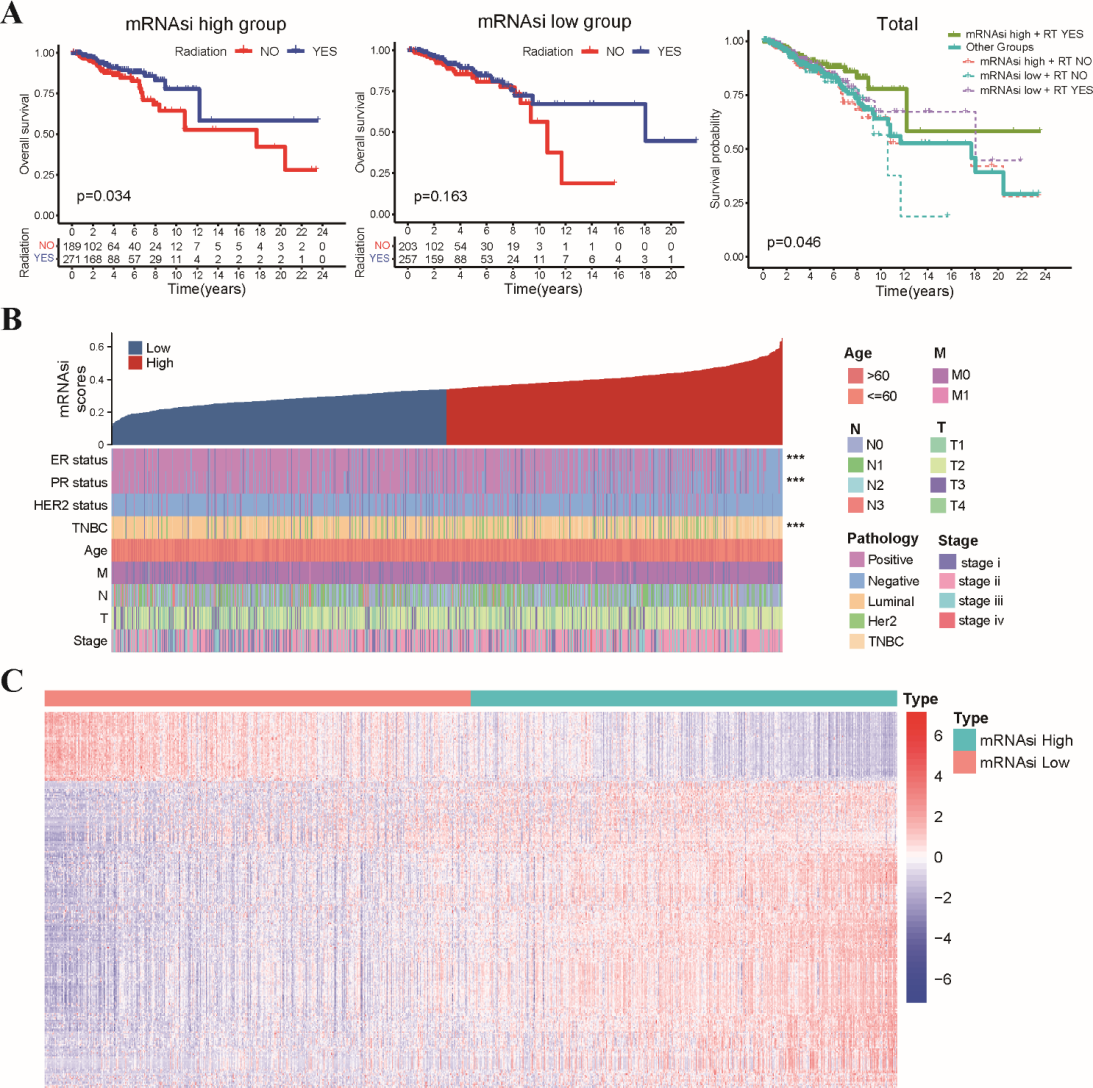
Association of mRNAsi scores with radiosensitivity, clinicopathological features, and stemness-related gene expression in TCGA-BRCA dataset. **(A)** The Kaplan-Meier curves depict OS for patients stratified by radiotherapy status and mRNAsi level. Patients in the high mRNAsi group who received radiotherapy exhibited significantly improved OS compared to those in other groups. **(B)** A summary of the association between mRNAsi scores and various clinicopathological characteristics of TCGA-BRCA patients. **(C)** The heatmap displays the differential expression levels of 267 identified SRGs between the high and low mRNAsi groups. *** p<0.001.

The study’s workflow was shown in **Supplementary Figure S1**. Stemness indices were calculated for TCGA-BRCA patients using their mRNA expression data. Kaplan-Meier survival analysis revealed no significant association between overall survival OS and mRNAsi scores across the entire TCGA-BRCA cohort (**Supplementary Figure S2A**). Interestingly, patients with high mRNAsi scores who received radiotherapy exhibited significantly improved OS compared to those who did not. Conversely, no significant difference in OS was observed between radiotherapy (RT) and non-radiotherapy (Non-RT) patients within the low mRNAsi group (**Figure 1A**). These findings suggested a potential interaction effect between mRNAsi and radiotherapy on patient prognosis. Next, we investigated the relationship between mRNAsi scores and clinicopathological features in TCGA-BRCA patients (**Figure 1B**). Patients in the low mRNAsi group displayed a significantly higher proportion of ER/PR-positive tumors compared to the high mRNAsi group (**Supplementary Figures S2B, C**). In addition, we performed differential gene expression analysis between the high and low mRNAsi groups. This analysis identified a total of 267 DEGs, designated as stemness-related genes (SRGs). Among these SRGs, 218 were upregulated and 49 were downregulated in the high mRNAsi group compared to the low mRNAsi group (**Figure 1C**).

### Construction of a radiosensitivity signature based on SRGs in the TCGA-BRCA dataset

**Figure 2:**
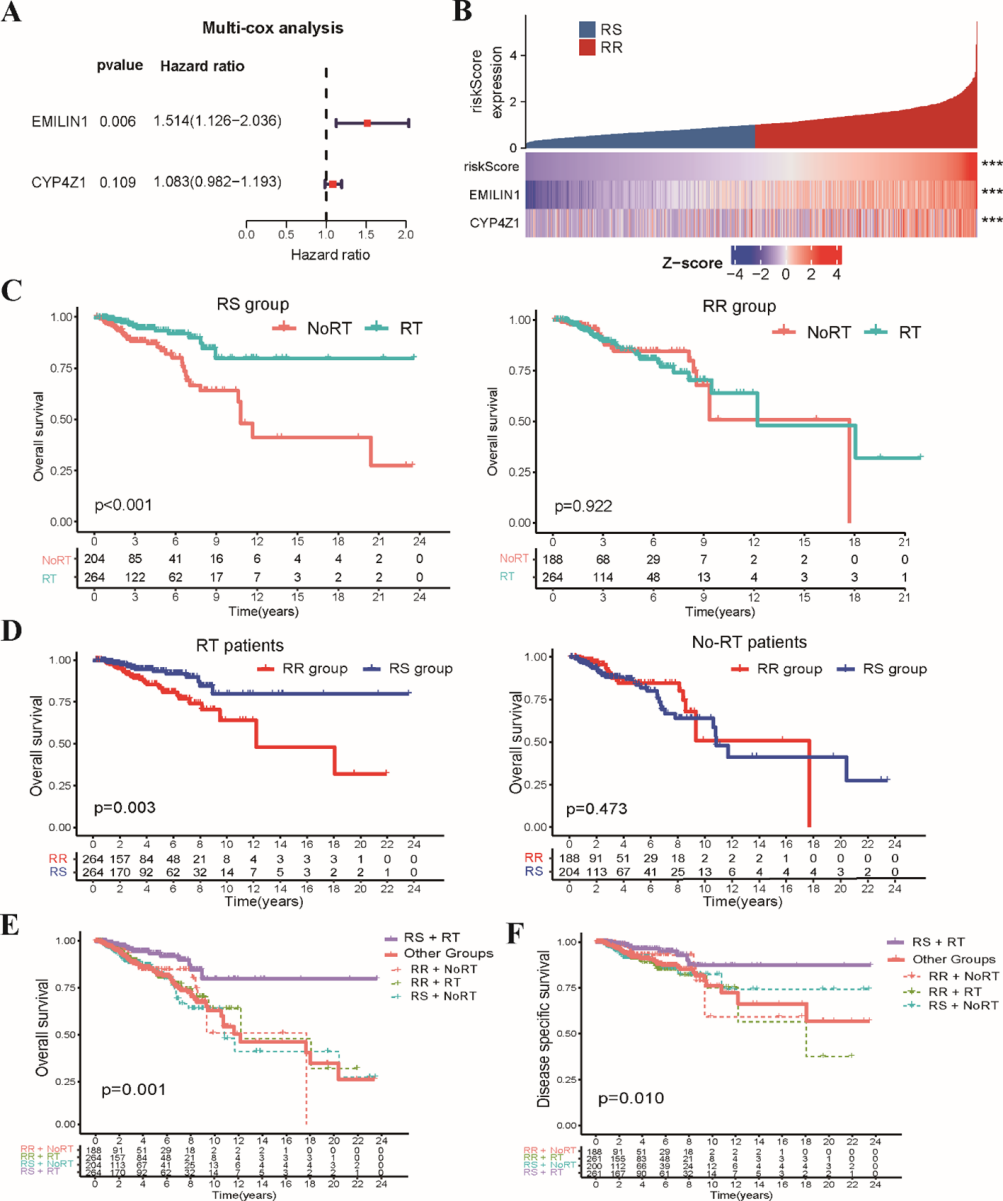
Development of the stemness-related radiosensitivity signature in the TCGA-BRCA dataset. **(A)** The forest plot depicts the hazard ratios and 95% confidence intervals for genes within the radiosensitivity signature using multivariate Cox analysis. **(B)** The heatmap visualizes the expression profiles of the two genes composing the radiosensitivity risk scores across RS and RR groups. **(C)** The Kaplan-Meier plot illustrates OS within each radiosensitivity group (RS/RR groups) for patients receiving radiotherapy compared to non-radiotherapy. **(D)** The Kaplan-Meier curves depicts the impact of radiosensitivity on OS stratified by radiotherapy status (RT/Non-RT patients). **(E)** The Kaplan-Meier curve demonstrates OS for RT patients in the RS group compared to other groups. **(F)** The Kaplan-Meier curve shows DSS for RT patients in the RS group compared to all other patients. *** p<0.001.


Subsequently, we constructed a clinically applicable radiosensitivity signature for predicting radiosensitivity in BRCA patients. Based on 267 SRGs identified in the TCGA-BRCA dataset, we performed univariate Cox regression analysis on the expression data of SRGs in both radiotherapy and non-radiotherapy patients. Our analysis revealed 15 SRGs that were significantly prognostic for OS in radiotherapy patients, but not in non-radiotherapy patients (**Supplementary Figures S3A–C**). Next, LASSO regression analysis was applied to the 15 prognostic SRGs identified in radiotherapy patients (**Supplementary Figure S3D**), which resulted in the selection of five key variables (**Supplementary Table S2**). These selected variables were subsequently used in the multivariate Cox regression analysis to establish the radiosensitivity signature. To ensure the validity of our Cox regression model, we conducted a PH assumption test for the 15 SRGs. **Supplementary Table S3** showed that both the individual and global P-values exceed 0.05, indicating that the hazard ratios are stable over time. These analyses identified two key genes, EMILIN1 and CYP4Z1, that were critical for establishing the radiosensitivity signature (**Figure 2A**). The formula for the radiosensitivity risk score is as follows:

Risk Score=(0.415×expression of EMILIN1)+(0.0795×expression of CYP4Z1).

To evaluate the prognostic potential of the radiosensitivity signature, we classified the entire TCGA-BRCA patients into RS and RR groups based on the median radiosensitivity risk score (**Figure 2B**). Kaplan-Meier survival analysis revealed a significant interaction effect between radiosensitivity status and radiotherapy on patient’s survival. Radiotherapy patients within the RS group exhibited significantly improved OS compared to non-radiotherapy patients. Conversely, no significant difference in OS was observed between radiotherapy and non-radiotherapy patients in the RR group (**Figure 2C**). Furthermore, within the radiotherapy patient population, those classified as RS displayed significantly improved OS compared to those in the RR group. No significant difference in OS was observed between RS and RR groups in the non-radiotherapy patients (**Figure 2D**). In general, radiotherapy patients in RS subgroup displayed significantly improved OS compared to those in the other groups (**Figure 2E**). Similar to the findings for OS, disease-specific survival (DSS) analysis revealed a significant benefit for radiotherapy in the RS group compared to the other groups (**Figure 2F**). These combined results support the potential of the radiosensitivity signature as a tool for predicting response to radiotherapy and potentially guiding treatment decisions in BRCA patients.

### Validation of the stemness-related radiosensitivity signature

**Figure 3:**
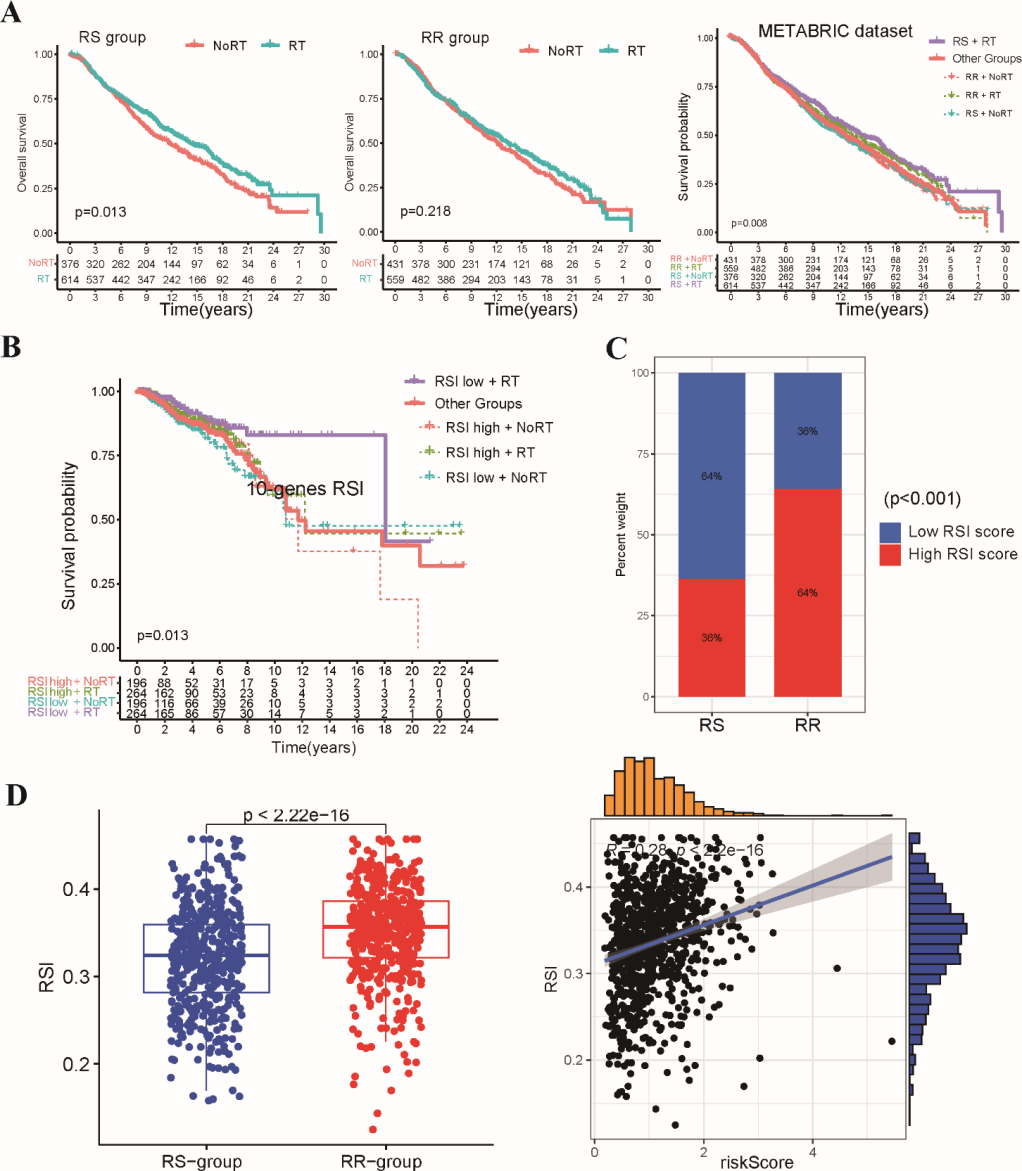
Validation of the stemness-related radiosensitivity signature. **(A)** The Kaplan-Meier curves depict OS for patients within the METABRIC dataset stratified by radiosensitivity (RS vs. RR) and radiotherapy status. **(B)** The Kaplan-Meier plot shows OS for radiotherapy patients within the TCGA-BRCA dataset categorized by the 10-gene RSI score (low vs. high). **(C)** The boxplot compares the distribution of 10-gene RSI scores in the RS and RR groups. **(D)** Correlation analysis between 10-gene RSI scores and radiosensitivity risk score in present study.

To assess the generalizability of the radiosensitivity signature, we performed external validation using the METABRIC-BRCA dataset. Patients in the METABRIC-BRCA dataset were classified into RS and RR groups using the same formula established in the TCGA-BRCA dataset. Consistent with our initial findings, Kaplan-Meier analysis revealed that radiotherapy patients within the RS group exhibited significantly improved OS compared to patients in other subgroups (**Figure 3A**). This finding suggests that the radiosensitivity signature may hold promise as a clinically applicable tool for predicting response to radiotherapy in BRCA patients beyond the TCGA-BRCA dataset.

We conducted a comparative analysis of our two-gene radiosensitivity signature with a previously proposed 10-gene RSI known to exhibit a close correlation with radiosensitivity in diverse cancer types (15). We calculated the 10-gene RSI for TCGA-BRCA patients using the linear regression algorithm proposed by Eschrich et al. (**Supplementary Table S3**). Our investigation revealed that radiotherapy patients who fell within the lower RSI group displayed significantly improved survival compared to those in other groups (**Figure 3B**). This result aligns with the established role of the 10-gene RSI signature in predicting response to radiotherapy. The distribution of patients across the RS and RR groups based on our signature mirrored the distribution based on the 10-gene RSI, with the RS group enriched for low-RSI patients and the RR group enriched for high-RSI patients (**Figure 3C**). In addition, we observed a positive correlation between our radiosensitivity risk score and the 10-gene RSI score in the TCGA-BRCA cohort (**Figure 3D**). Overall, the results from the validation cohort and the comparison with the existing signature support the potential of our radiosensitivity signature as a biomarker for predicting response to radiotherapy in BRCA patients.

### Tumor characteristics and functional enrichment analysis of RS and RR patients

**Figure 4:**
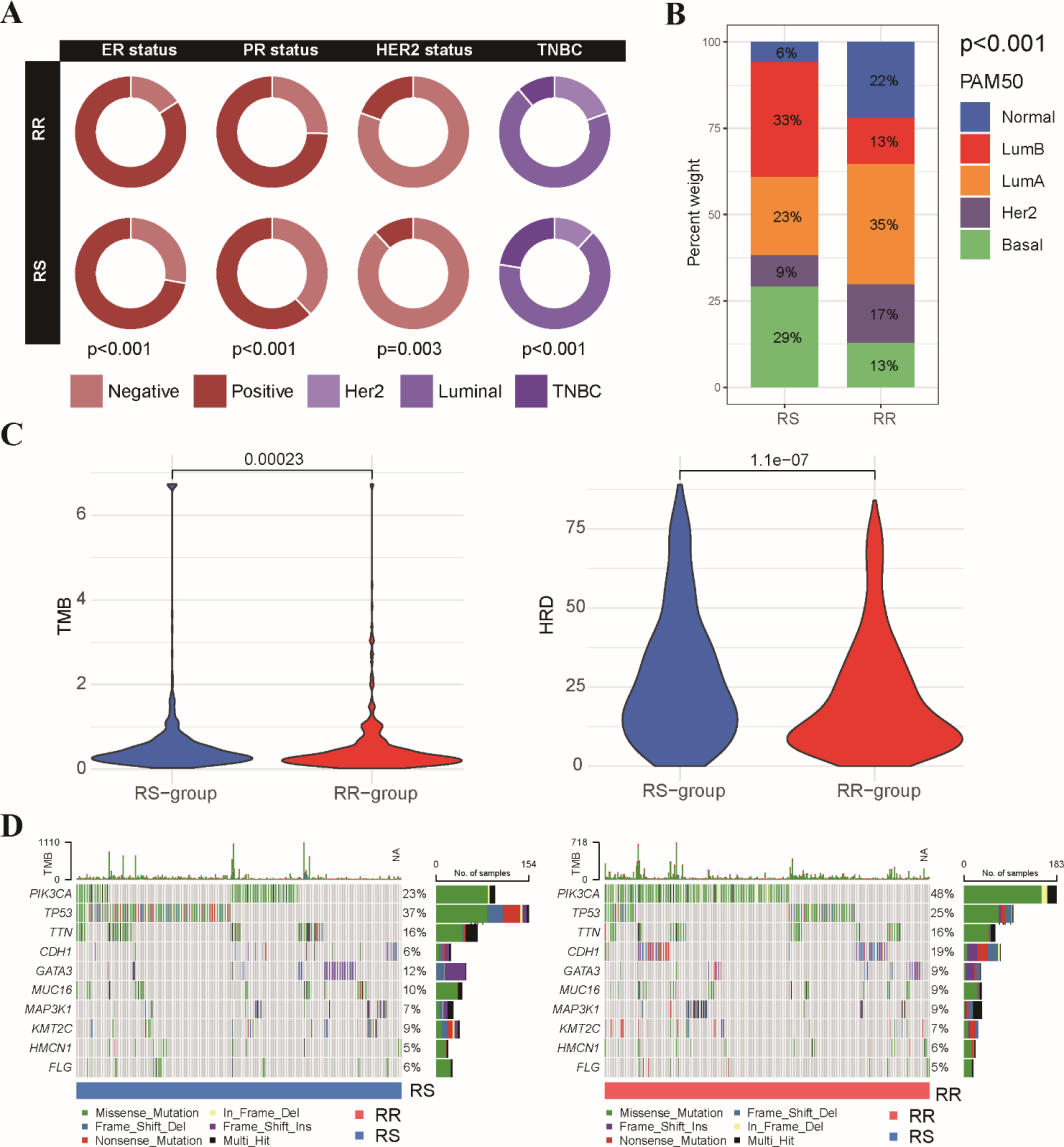
Association between radiosensitivity signature and pathological features in BRCA patients. **(A)** The circus plot depicts the prevalence of ER, PR, and HER2 positivity within the RS and RR groups. **(B)** The stacked bar chart illustrates the proportion of patients with different breast cancer PAM50 subtypes (basal, luminal A, luminal B, HER2-enriched, and normal-like) within the RS and RR groups. **(C)** The violin plot shows the distribution of tumor mutational burden (TMB) values and HRD scores in the RS and RR groups. **(D)** The waterfall plots depict the top 10 most frequently mutated genes in both the RS and RR groups.

We compared the clinical characteristics of patients within the RS and RR groups identified based on the radiosensitivity signature (**Supplementary Table S4**). We further evaluated the distribution of hormone receptor (HR) and HER2 status between the RS and RR groups. RR tumors exhibited a significantly higher prevalence of estrogen receptor (ER), progesterone receptor (PR) and Her2 positivity compared to RS tumors (**Figure 4A**). Analysis of breast cancer PAM50 subtype revealed an enrichment of Basal and Luminal-B subtypes within the RS group, while Luminal-A and Normal subtypes were more prevalent in the RR group (**Figure 4B**). We proceeded to examined the association between the radiosensitivity signature and genomic alterations. Analysis of tumor mutation burden (TMB) and homologous recombination deficiency (HRD) score revealed a significant association with the radiosensitivity signature. Patients in the RS group displayed higher HRD scores, potentially indicating increased reliance on DNA repair pathways (**Figure 4C**). We also generated a comprehensive mutational profile, identifying the top 10 mutated genes across both groups. Missense mutations were the most frequent mutation type observed. Interestingly, PIK3CA mutations were more prevalent in the RR group, while TP53 mutations were enriched in the RS group (**Figure 4D**).

**Figure 5:**
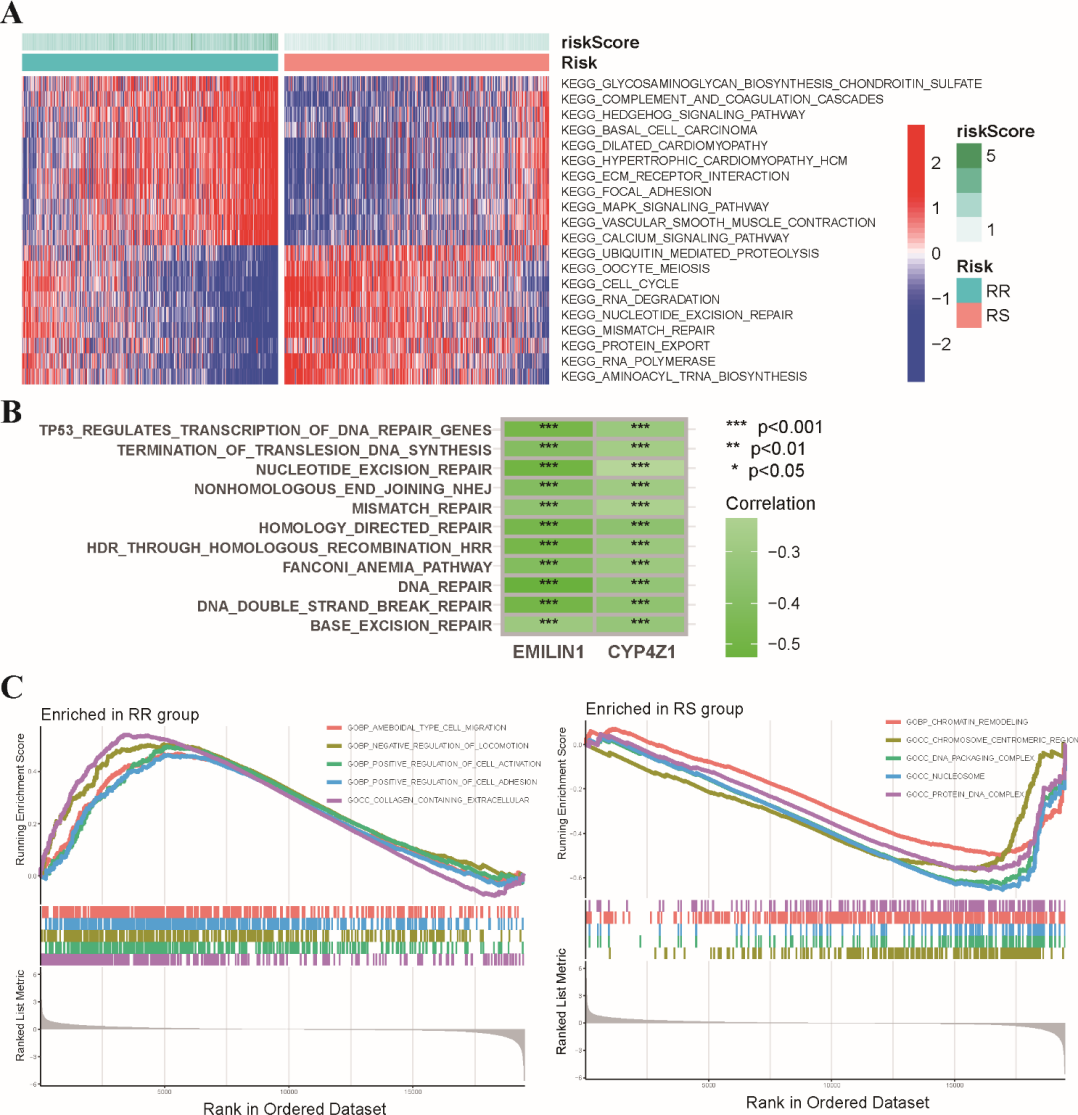
Functional enrichment analysis of the radiosensitivity signature in BRCA patients. **(A)** The heatmap visualizes the enrichment scores of the top differentially enriched hallmark pathways between the RS and RR groups. **(B)** Analysis of the coordinated expression patterns between two signature genes and hallmark DNA damage repair pathways. **(C)** This plot depicts the results of GSEA, highlighting the biological functions associated with the RS and RR groups in BRCA patients. * p<0.05, ** p<0.01, *** p<0.001.

We further explored the molecular pathways associated with the radiosensitivity signature. This analysis revealed significant enrichment of DNA damage repair pathways, including mismatch repair, nucleotide excision repair, and cell cycle regulation pathways in the RS group. Conversely, the RR group exhibited enrichment in the MAPK signaling pathway and the Hedgehog signaling pathway (**Figure 5A**). We further performed a correlation analysis examining the expression levels of two signature genes with hallmark DNA damage repair pathways in BRCA (**Figure 5B**). In addition, GSEA demonstrated that the RR group displayed enrichment in pathways related to positive regulation of cell activation and adhesion, while the RS group exhibited a stronger association with chromatin remodeling and DNA packaging complex pathways (**Figure 5C**). These findings suggest that the radiosensitivity signature may reflect underlying differences in DNA repair mechanisms potentially influencing response to radiotherapy.

### Tumor immune infiltration characteristics of the radiosensitivity signature

**Figure 6:**
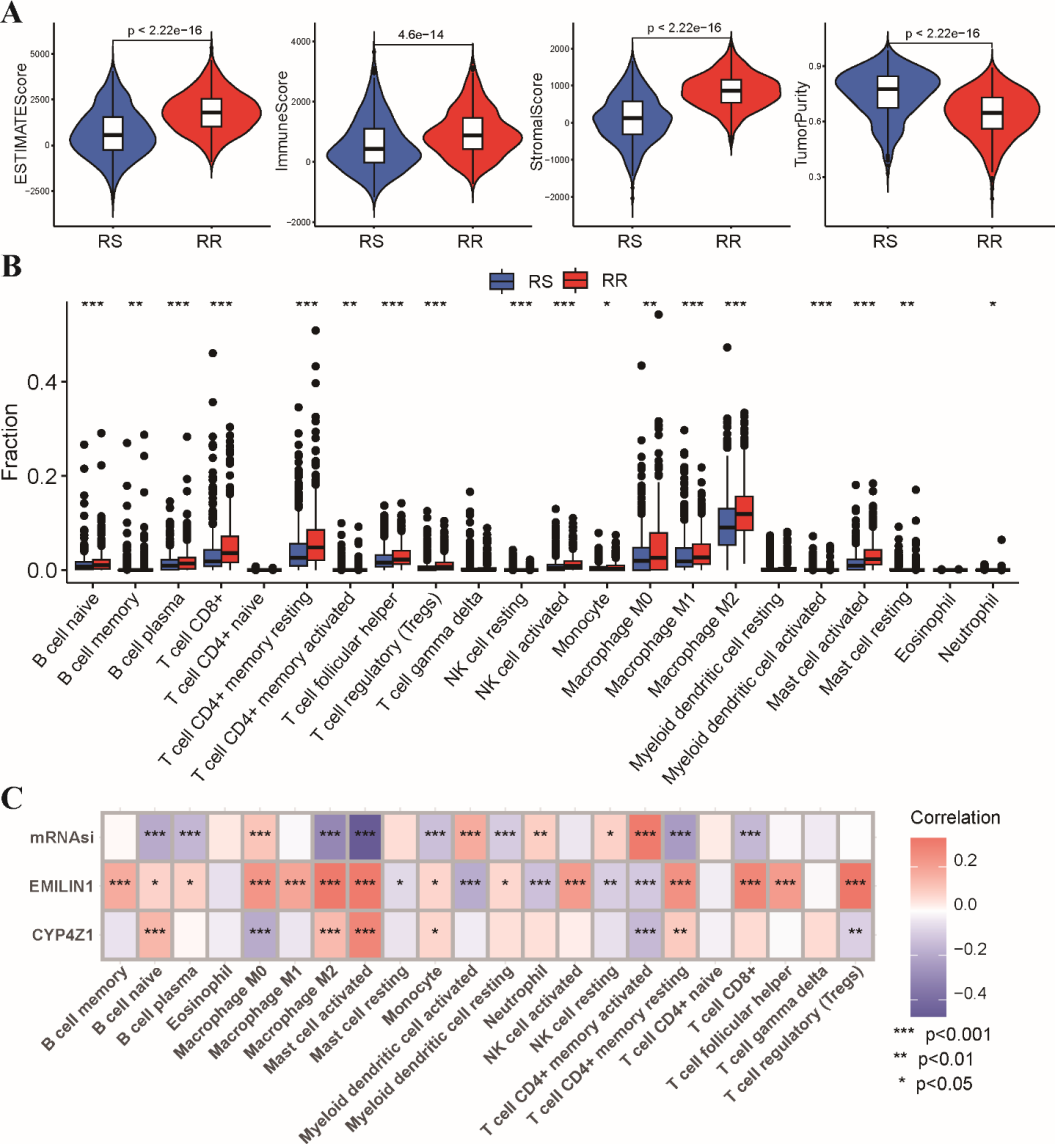
Tumor immune microenvironment analysis in RS and RR groups. **(A)** The violin plots depict the estimated score, stromal score, immune score, and tumor purity within the RS and RR groups. **(B)** The bar chart shows the relative abundance of 22 estimated immune cell types in the RS and RR groups, as determined by CIBERSORT analysis. **(C)** The correlation coefficients between the expression levels of the two genes in the radiosensitivity signature and the estimated abundance of 22 immune cell types. * p<0.05, ** p<0.01, *** p<0.001.

Growing evidence suggests that the tumor immune microenvironment (TIME) plays a critical role in response to radiotherapy (26, 27). To investigate potential differences in the immune landscape, we employed the ESTIMATE algorithm to assess immune and stromal components within the tumor microenvironment. This analysis revealed significantly higher estimated scores, stromal scores, and immune scores in the RR group compared to the RS group. Conversely, tumor purity was significantly lower in the RS group (**Figure 6A**). Next, we evaluated the composition of tumor-infiltrating immune cells using CIBERSORT analysis. This analysis identified a higher abundance of CD8+ T cells, regulatory T cells (Tregs), and M1/M2 macrophages within the RR group. In contrast, RS group exhibited a higher level of myeloid dendritic cells and activated memory CD4 T cells compared to the RS group (**Figure 6B**). We performed correlation analysis to further explore potential relationships between the radiosensitivity signature genes and immune cell infiltration (**Figure 6C**). These results further strengthening the link between the radiosensitivity signature and the tumor immune microenvironment.

### Association between the radiosensitivity signature and anti-tumor therapy

**Figure 7:**
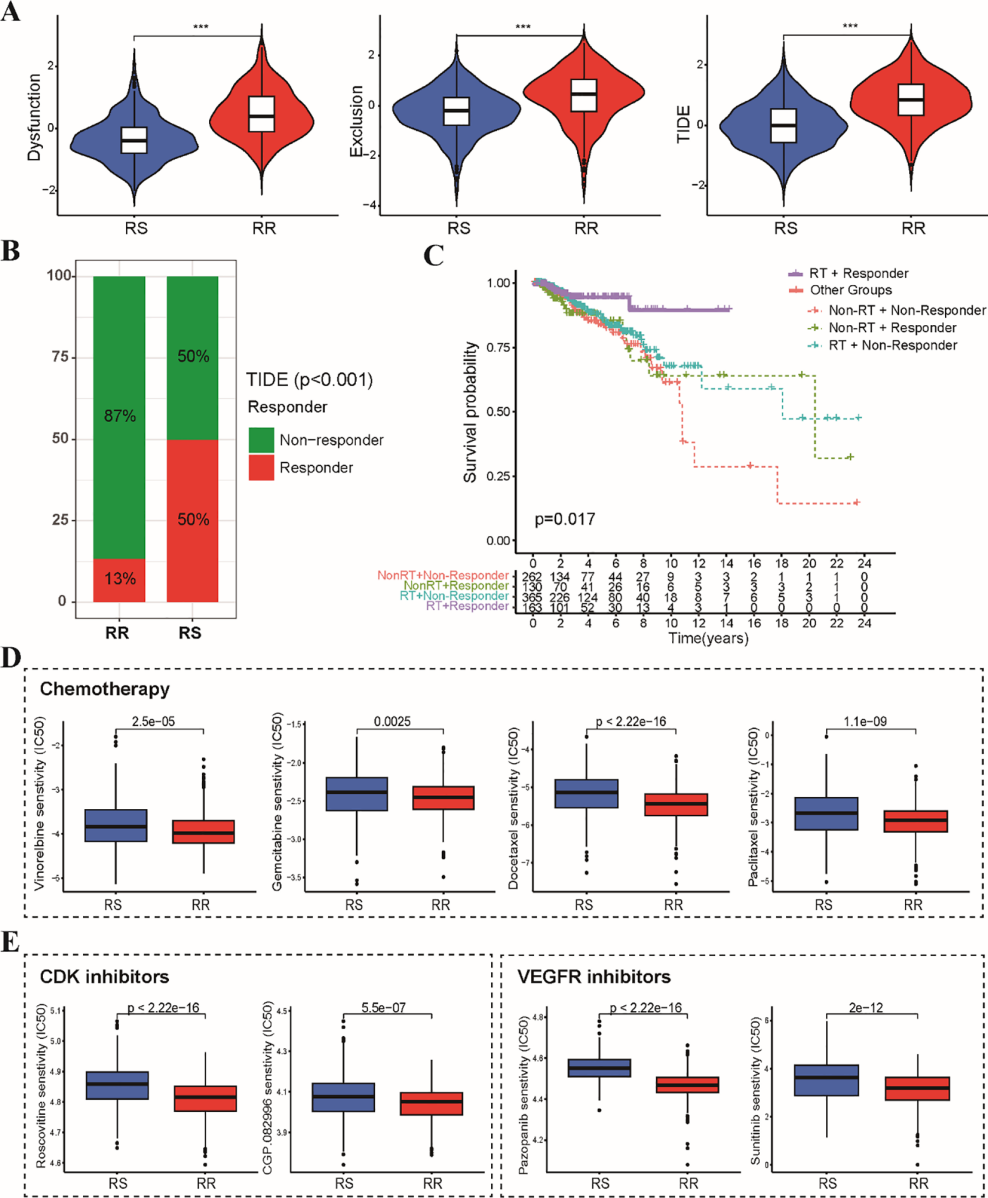
Sensitivity analysis of immunotherapy, chemotherapy and targeted therapy between RS and RR groups. **(A)** The violin plots depict the T cell exclusion, T cell dysfunction, and TIDE scores within the RS and RR groups. **(B)** The stacked bar chart illustrates the proportion of patients classified as responders and non-responders to immunotherapy within the RS and RR groups. **(C)** The Kaplan-Meier curve demonstrates that radiotherapy patients in the responder group exhibited better prognosis compared to all other patients. **(D)** The boxplots depict the IC50 values for first-line chemotherapeutic drugs (paclitaxel, docetaxel, vinorelbine, and gemcitabine) in the RS and RR groups. **(E)** The boxplots show the difference in IC50 values for CDK and VEGFR kinase inhibitors between the RS and RR groups. *** p<0.001.

To explore the potential impact of the radiosensitivity signature on response to various anti-tumor therapies, we investigated the association with immunotherapy, chemotherapy, and targeted therapy. Analysis using the TIDE algorithm revealed lower TIDE scores, T cell exclusion and T cell dysfunction scores in the RS group compared to the RR group (**Figure 7A**). This indicates that BRCA patients within the RS group displayed a significantly better response to immunotherapy compared to the RR group (**Figure 7B**). Furthermore, analysis stratified by radiotherapy status revealed that patients who received radiotherapy in responder group exhibited better prognosis in comparison to other subgroups (**Figure 7C**). Sensitivity analysis demonstrated that the RR group exhibited lower IC50 values for first-line chemotherapy drugs, including paclitaxel, docetaxel, vinorelbine, and gemcitabine. This suggests that patients in the RR group may be more sensitive to these chemotherapeutic agents (**Figure 7D**). Estimation of IC50 values for targeted therapy drugs indicated that the RS group was less sensitive to CDK inhibitors and VEGFR kinase inhibitors (**Figure 7E**). These findings suggest that CDK and VEGFR inhibitors might be potential candidates to overcome resistance to radiotherapy in the RR group.

### PD-L1 expression stratifies radiosensitivity and predicts immunotherapy response in BRCA

**Figure 8:**
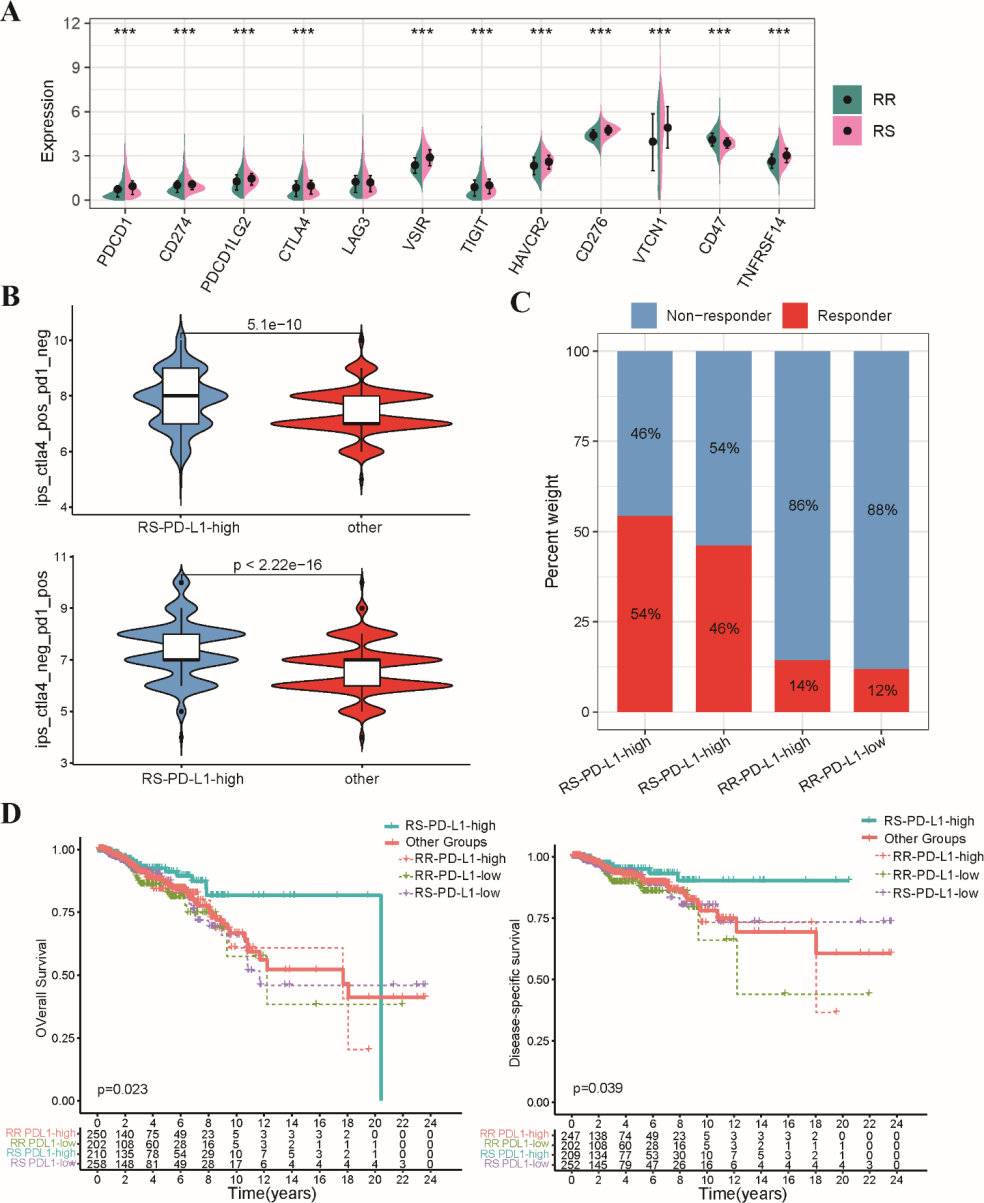
Analysis of relationship between the radiosensitivity signature and PD-L1 expression. **(A)** The bar chart depicts the expression levels of various immune checkpoint genes in the RS and RR groups. **(B)** The violin plot illustrates the distribution of IPS scores for both CTLA-4 and PD-1 inhibitors within the RS-PD-L1-high and other groups. **(C)** The stacked bar chart visualizes the proportion of patients classified as responders and non-responders to immunotherapy within the RS-PD-L1-high and other groups. **(D)** This Kaplan-Meier plot shows OS and DSS for patients categorized as RS-PD-L1-high and all other groups. *** p<0.001.

Our study investigated the relationship between a radiosensitivity signature and immune checkpoint expression in BRCA patients. We observed a general upregulation of immune checkpoint genes, including PD-L1, in the RS group compared to the RR group (**Figure 8A**). Patients were categorized into two groups according to their median levels of PD-L1 expression, with the RS group that had high PD-L1 levels defined as the RS-PDL1-high subgroup, while the remaining patients were classified into the “Other” group. The ESTIMATE analysis revealed enrichment of estimate scores, stromal scores, and immune scores in the RS-PD-L1-high subgroup (**Supplementary Figure S4A**). Furthermore, CIBERSORT revealed a heightened infiltration of CD8+ T cells, CD4+ T cells, and other immune cell types within the TIME of the RS-PD-L1-high subgroup, suggesting an immunologically active (**Supplementary Figure S4B**). Given the established significance of PD-L1 for immunotherapy response, we further explored its association with the radiosensitivity signature. The Immunophenoscore (IPS) algorithm predicted a better response to both PD-1 and CTLA-4 inhibitors within the RS-PD-L1-high group (**Figure 8B**). Consistent with these observations, TIDE algorithm predicted a more favorable response to immunotherapy in RS-PD-L1-high group (**Figure 8C**). This association translated to improved OS and DSS for patients in the RS-PD-L1-high subgroup compared to other groups (**Figure 8D**). These findings support the notion that PD-L1 expression can influence immunotherapeutic response within the context of radiosensitivity, highlighting the potential for combining these factors for personalized treatment strategies in BRCA patients.

### Validation of the two radiosensitivity genes *in vitro* experiments

**Figure 9:**
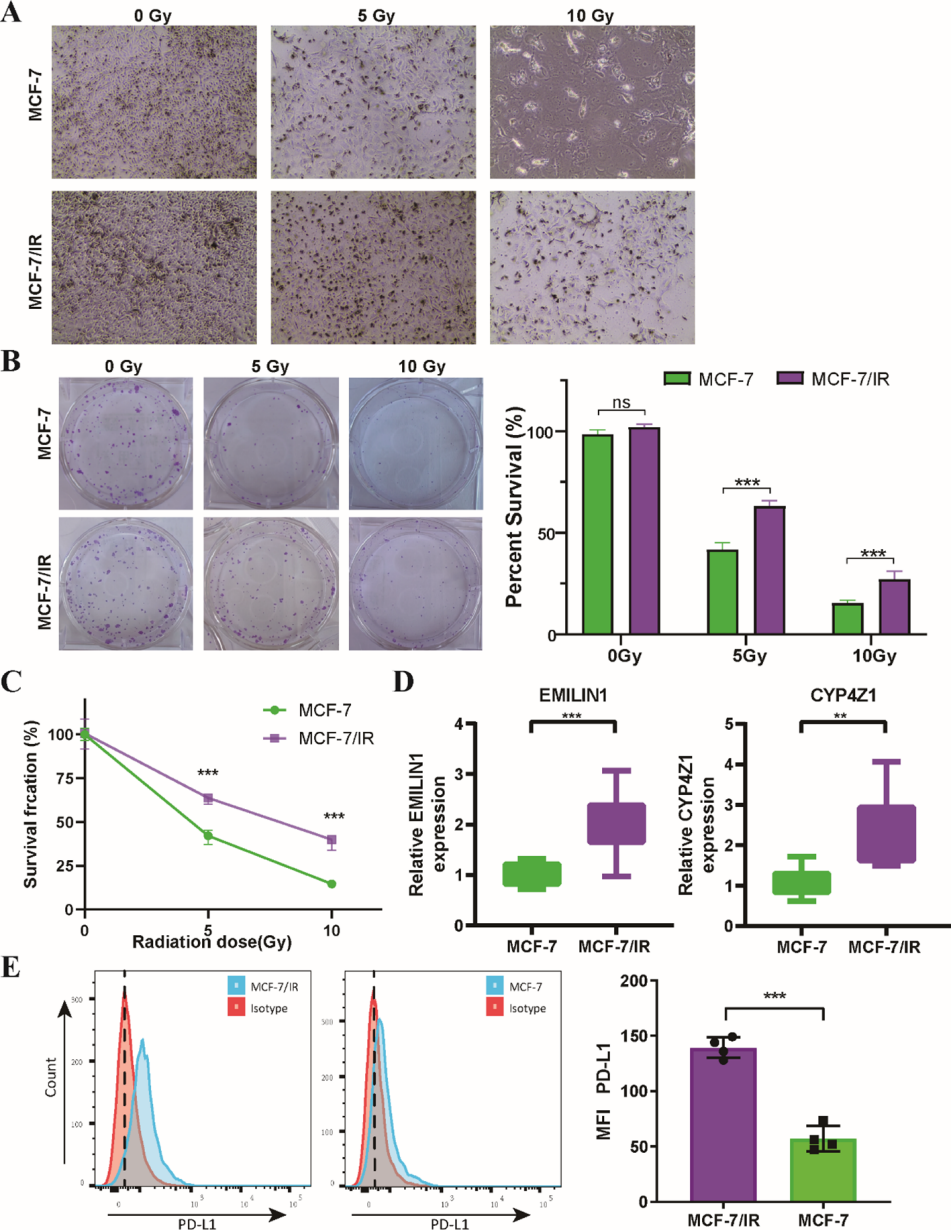
*In Vitro* validation of the two radiosensitivity genes. **(A)** Microscopy images showing MCF-7 and MCF-7/IR cells cultured for 48 hours following radiation treatment. **(B)** Clonogenic survival of MCF-7 and MCF-7/IR cells after irradiation with 5 and 10 Gy. **(C)** Proliferative capacity Proliferative capacity of MCF-7 and MCF-7/IR cells following ionizing radiation exposure, as measured by the CCK-8 assay. **(D)** Relative expression levels of EMILIN1 and CYP4Z1 in MCF-7 and MCF-7/IR cells, determined by qRT-PCR. Both genes displayed significantly higher expression in MCF-7/IR cells compared to MCF-7 cells. **(E)** Histograms of PD-L1 expression on MCF-7 and MCF-7/IR cells *in vitro*. ** p<0.01. *** p<0.001. ns, not significant.

We employed *in vitro* experiments to further validate the predictive power of the radiosensitivity signature identified through bioinformatic analysis. We leveraged two well-characterized breast cancer cell lines: MCF-7, known for its radiosensitive phenotype (28, 29), and MCF-7/IR, a derivative exhibiting radio-resistance (**Figure 9A**). The clonogenic assay results showed a marked decrease in the ability of MCF-7 cells to form colonies after radiation exposure, in contrast to MCF-7/IR cells, which exhibited a greater capacity for colony formation. Notably, the colonies generated by MCF-7/IR cells were not only more numerous but also larger in size compared to those formed by MCF-7 cells, suggesting a greater degree of radio-resistance (**Figure 9B**). Furthermore, the proliferative capacity of both cell lines following ionizing radiation exposure was assessed using the validated CCK-8 assay. As anticipated, MCF-7/IR cells displayed significantly higher viability compared to MCF-7 cells across all radiation doses (**Figure 9C**). Consistent with our bioinformatics analysis, the expression levels of EMILIN1 and CYP4Z1 were demonstrably elevated in the radioresistant MCF-7/IR cells compared to their radiosensitive MCF-7 counterparts (**Figure 9D**). Interestingly, our results indicate that PD-L1 expression is elevated in irradiated MCF-7/IR cells compared to MCF-7 cells, suggesting a potential link between radio-resistance and immune checkpoint expression (**Figure 9E**). These findings provide initial *in vitro* validation for the proposed two-gene signature, suggesting its potential utility in predicting cellular response to radiotherapy.

## Discussion

The emergence of radiotherapy resistance remains a formidable challenge in BRCA therapy (30). Accumulating evidence point to a subpopulation of CSCs, characterized by their stem-like properties, are believed to contribute to radiation resistance, leading to patient relapse and mortality (31). Therefore, a deeper understanding of how cancer stemness influences radiosensitivity in BRCA is crucial for overcoming radio-resistance and developing strategies against cancer stemness. Our study identified a positive correlation between the mRNAsi score and patient prognosis following radiotherapy. While it is well-documented that a higher proportion of CSCs is generally associated with increased radioresistance and poorer patient outcomes (32), our findings suggest a more complex relationship. Specifically, the high mRNAsi group, which reflects greater stemness characteristics, exhibited improved OS following radiotherapy. This observation may indicate that the presence of certain CSC populations, in conjunction with a favorable tumor immune microenvironment, can lead to enhanced treatment responses. The distinct immune profiles observed in the high mRNAsi group suggest that these tumors may be more responsive to radiotherapy-induced immune activation, thereby improving patient outcomes despite the inherent challenges posed by CSCs. Building upon this finding, we constructed a distinct radiosensitivity signature by integrating stemness-related genes and demonstrated its effectiveness in stratifying patients into RS and RR groups. This stratification revealed significant differences in response to radiotherapy, highlighting the potential of the signature to capture inter-tumor heterogeneity in radiosensitivity within the BRCA population.

High-throughput molecular profiling has emerged as a powerful tool for unraveling the complexities of individual radiosensitivity (33). This approach enables the construction of gene signatures that can predict patient responses to radiotherapy. From a clinical perspective, radiosensitivity can be defined using the following criteria: (1) in the absence of radiotherapy, both RS and RR groups exhibit similar survival rates. (2) upon receiving radiotherapy, the RS group experiences significantly improved survival outcomes compared to the RR group (34). Our study presents a novel prediction model for breast cancer radiosensitivity based on a two-gene signature composed of stemness-related genes. The signature was evaluated to ensure they meet established criteria for radiosensitivity. Notably, the signature was further validated using the METABRIC dataset. Moreover, we compared our model with existing pan-cancer radiosensitivity study. While subgroup analysis of the TCGA-BRCA dataset revealed consistent results, the 10-gene RSI demonstrated superior sensitivity and specificity in predicting radiosensitivity. These findings collectively support the notion that our two-gene signature functions as an independent predictor of radiosensitivity for BRCA patients.

The clinical utility of harnessing a patient’s own immune system has emerged as a new pillar of anti-tumor therapy, including the use of radiotherapy (35). Compelling evidence underscores the critical role of the tumor immune microenvironment in influencing response to radiotherapy (36). Our analysis revealed significantly higher ESTIMATE scores, immune scores and higher abundance of CD8 T cells, regulatory T cells (Tregs), and M1/M2 macrophages within the RR group compared to the RS group. These observations suggest a more immunosuppressive microenvironment within RS tumors. Intriguingly, however, our data suggests that the RS group may be more susceptible to immunotherapy. This apparent paradox can be explained by the underlying mechanisms of immune checkpoint blockade therapy. Immunotherapy targets specific inhibitory receptors, such as PD-L1, that act as brakes on the immune system, preventing T cells from effectively recognizing and eliminating cancer cells (37, 38). These inhibitory receptors are often upregulated in immunosuppressive tumor microenvironments. By blocking these inhibitory signals, immune checkpoint blockade therapy can reinvigorate anti-tumor immune responses, even in settings characterized by immune suppression (39). Indeed, we observed a general upregulation of immune checkpoint genes, including PD-L1, in the RS group compared to the RR group. This finding suggests the presence of a potentially functional T cell population within the RS tumors that can be unleashed by immune checkpoint blockade. Therefore, the immunosuppressive microenvironment in RS tumors can be seen as a “double-edged sword.” While it contributes to tumor immune evasion, it also indicates the presence of a potentially functional immune system that can be reawakened by immune checkpoint blockade therapy. Collectively, these findings suggest that the radiosensitivity signature may not only predict response to radiotherapy but also hold promise as a biomarker for identifying patients who might benefit from immunotherapy.

To reverse radio-resistance in breast cancer, various chemotherapeutic and targeted agents are currently under investigation. The sensitivity analysis indicated that the RR group exhibited lower IC50 values for first-line chemotherapy drugs, suggesting an increased sensitivity to these treatments. This finding implies that patients in the RR group may derive significant benefits from these chemotherapeutic agents, potentially resulting in enhanced treatment outcomes. In contrast, the RS group showed diminished sensitivity to CDK inhibitors and VEGFR kinase inhibitors, revealing a therapeutic gap that could be addressed to counteract resistance to radiotherapy. Notably, VEGF has been linked to the development of abnormal tumor vasculature within the tumor microenvironment, which can lead to hypoxia (40). Tumor hypoxia has been demonstrated to diminish radiation sensitivity across various cancer types (41), which may partially explain the RR group’s sensitivity to VEGFR kinase inhibitors. Further investigation into the mechanisms underlying these sensitivities, including the role of specific genetic alterations and signaling pathways, will be essential for optimizing therapeutic approaches and improving patient outcomes in BRCA patients.

Our study identified two critical genes, EMILIN1 and CYP4Z1, that contribute significantly to the radiosensitivity signature in BRCA. EMILIN1, an extracellular matrix (ECM) glycoprotein, has been implicated in cancer progression and metastasis (42). EMILIN1 associates with elastic fibers, influencing tumor development (43). Previous studies have reported decreased EMILIN1 expression in metastatic BRCA tumors, suggesting a potential tumor-suppressive role (44). Our findings revealed a positive correlation between EMILIN1 expression and risk score, alongside upregulation in the RR group. As accumulating evidence suggests that interactions between the extracellular matrix and cancer cells can modulate radiosensitivity (45, 46). Our observations suggest a potential mechanism by which EMILIN1 might influence the ECM pathway and contribute to radio-resistance in RR group. Our study also identified CYP4Z1, a novel member of the CYP4 family, as upregulated in the RR group. CYP4Z1 is known for its overexpression in BRCA, often associated with high-grade tumors and a poorer overall prognosis (47). Functional studies have demonstrated that CYP4Z1 overexpression promotes tumor angiogenesis in breast cancer through the regulation of VEGF-A and TIMP-2 expression, potentially mediated by PI3K and ERK1/2 activation (48). Furthermore, CYP4Z1 expression has been shown to inhibit apoptosis, induce stemness, and contribute to chemotherapy insensitivity in BRCA (49). These findings, coupled with the established pro-angiogenic properties of CYP4Z1, provide compelling evidence for a close relationship between CYP4Z1 and radiosensitivity in BRCA.

A growing body of research is investigating the intricate link between stemness characteristics and radiosensitivity in different types of cancer. This study presents the first identification of a stemness-related radiosensitivity signature in BRCA patients. However, inherent limitations associated with the study design warrant further exploration. Firstly, the retrospective design, with its potential for selection bias and limitations on causality, and the reliance on bioinformatic analyses are inherent limitations of this study. Prospective validation in a clinical cohort is crucial to confirm the signature’s generalizability. Secondly, the study focused solely on the validation of the core genes, EMILIN1 and CYP4Z1. To gain a deeper understanding of the functional significance of the identified stemness-related genes in BRCA radiosensitivity, further validation through *in vitro* and *in vivo* models is necessary. Furthermore, while this study identifies a radiosensitivity signature, the development of a fully validated predictive system with defined cutoff values for clinical application requires further investigation, including prospective studies with larger cohorts and rigorous clinical validation.

## Conclusion

In conclusion, our study established a radiosensitivity signature based on stemness-related genes, potentially guiding treatment decisions in BRCA. BRCA patients in the RS group displayed a significantly better response to immunotherapy compared to the RR group, suggesting interplay between stemness, radiosensitivity, and immunotherapy efficacy. Additionally, the RR group may be more sensitive to CDK inhibitors, VEGFR kinase inhibitors, and potentially other chemotherapeutic drugs. Further investigation is needed to elucidate mechanisms and identify anti-tumor drugs synergistic with radiotherapy, paving the way for personalized treatment regimens based on the radiosensitivity signature.

## Data Availability

The original contributions presented in the study are included in the article/**Supplementary Material**. Further inquiries can be directed to the corresponding author/s.
