# Supplementary material for: Stemness-related gene signatures as a predictive tool for breast cancer radiosensitivity
Source: Front Immunol. 2025 Jan 31;16:1536284. doi: 10.3389/fimmu.2025.1536284 (PMC11825753; doi:10.3389/fimmu.2025.1536284)
Supplement: Supplementary file 1 [file DataSheet1.pdf]

Supplement Figures

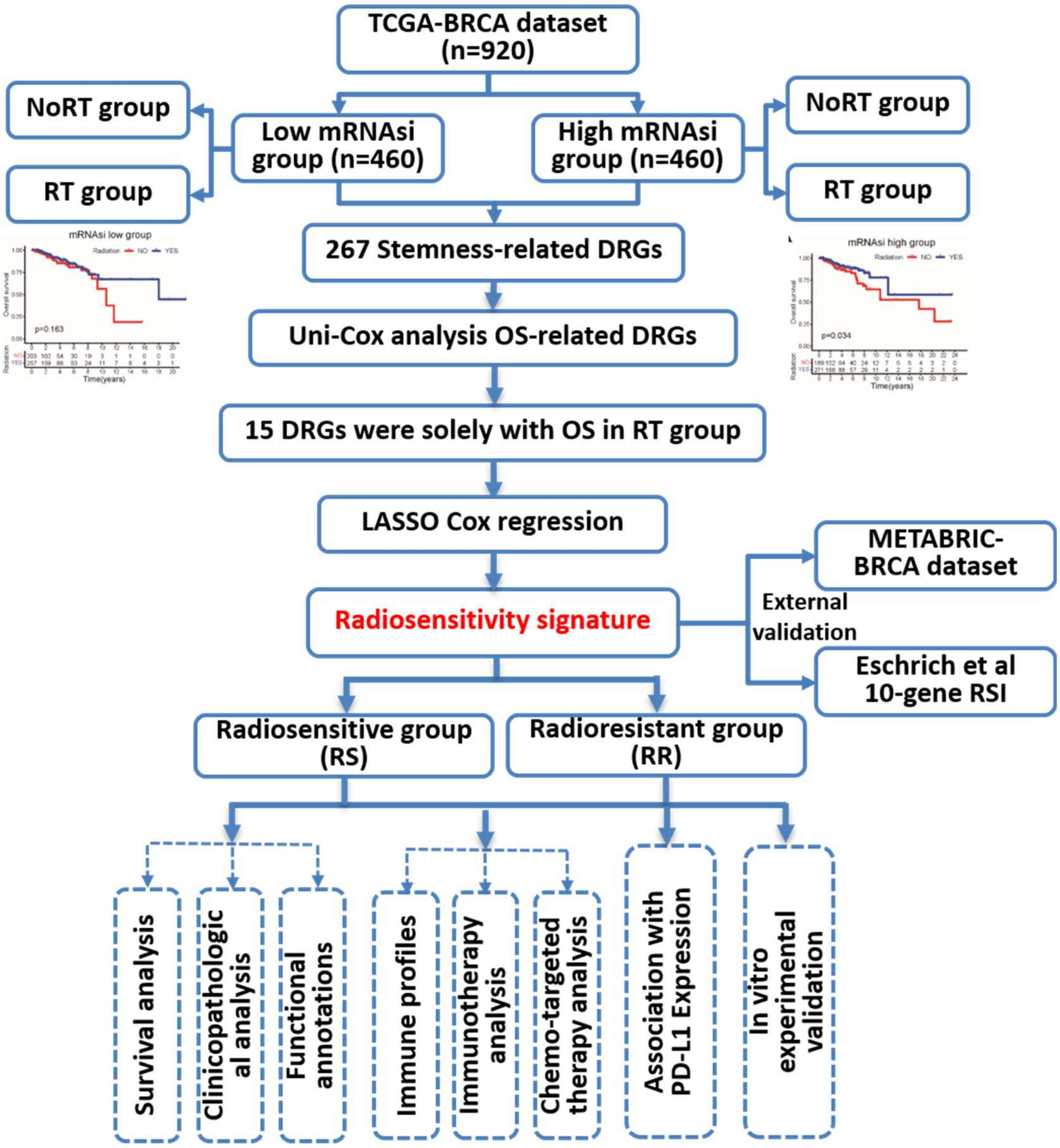

FIGURE S1 The study's workflow

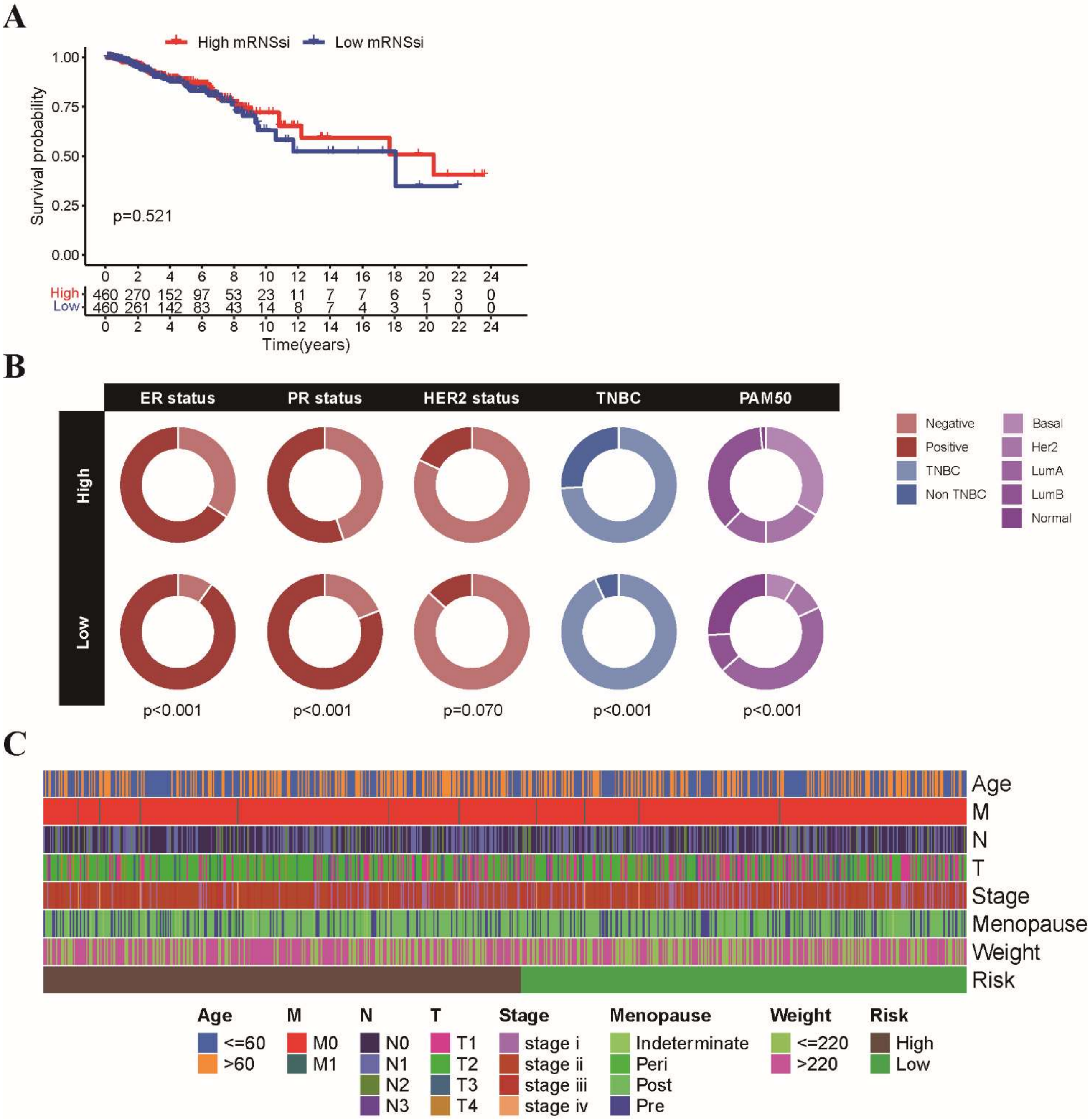

FIGURE S2 (A) Kaplan-Meier survival curves showing the OS outcomes of patients in the high and low mRNAsi group. **(B)** The circus plot visually represented the association between radiosensitivity cluster and the status of ER/PR and Her2 in breast cancer. **(C)** An overview of the relationship between the radiosensitivity cluster and clinicopathological parameters in patients with BRCA.

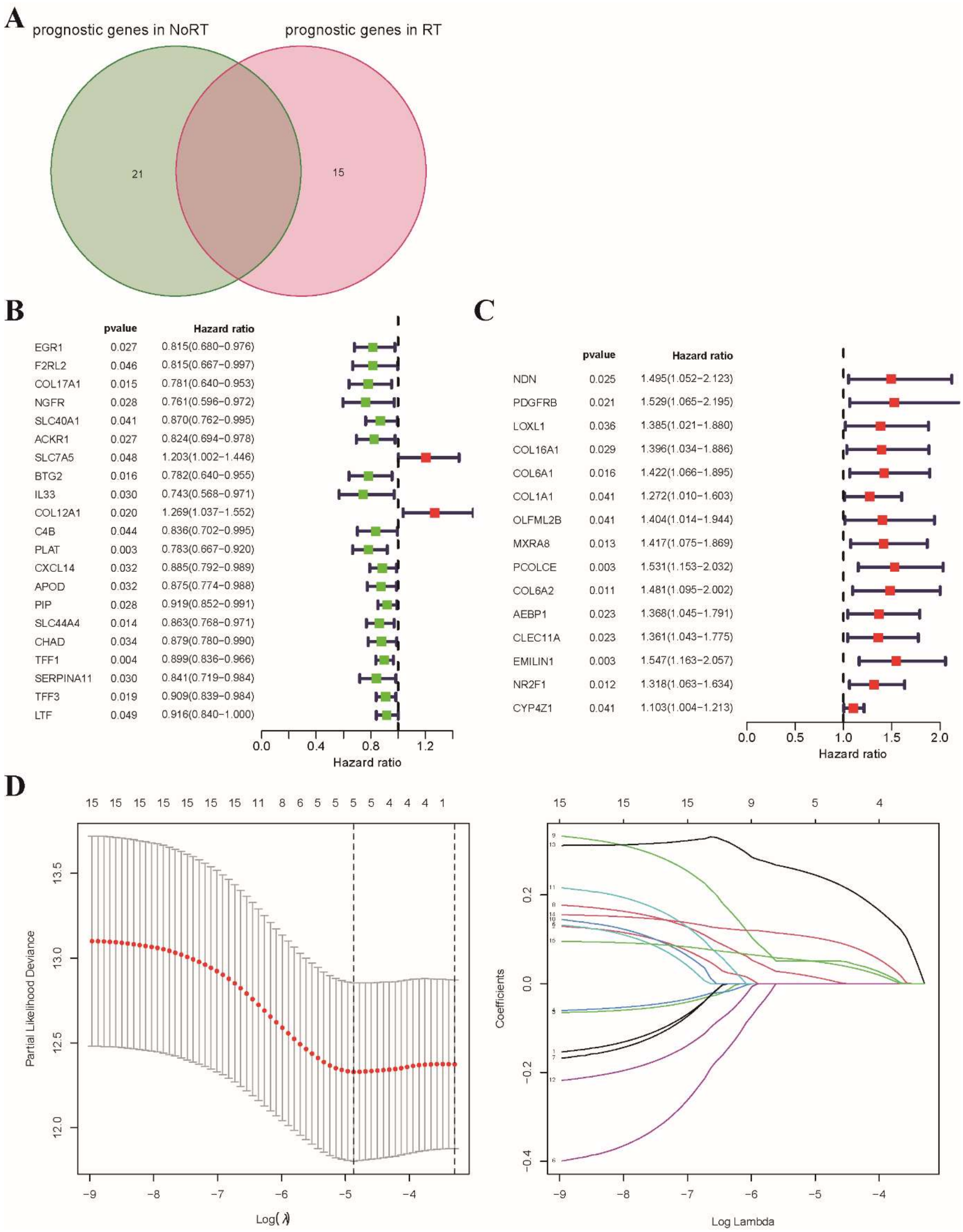

FIGURE S3 (A) Venn diagram showed that 15 stemness-related genes were significantly associated with OS in radiotherapy patients, but not in non-radiotherapy patients. **(B)** Forest plot displaying the results of the univariate Cox regression analysis conducted in the cohort of radiotherapy group and **(C)** non-radiotherapy group. **(D)** The least absolute shrinkage and selection operator (LASSO) method of SRGs associated with prognosis.

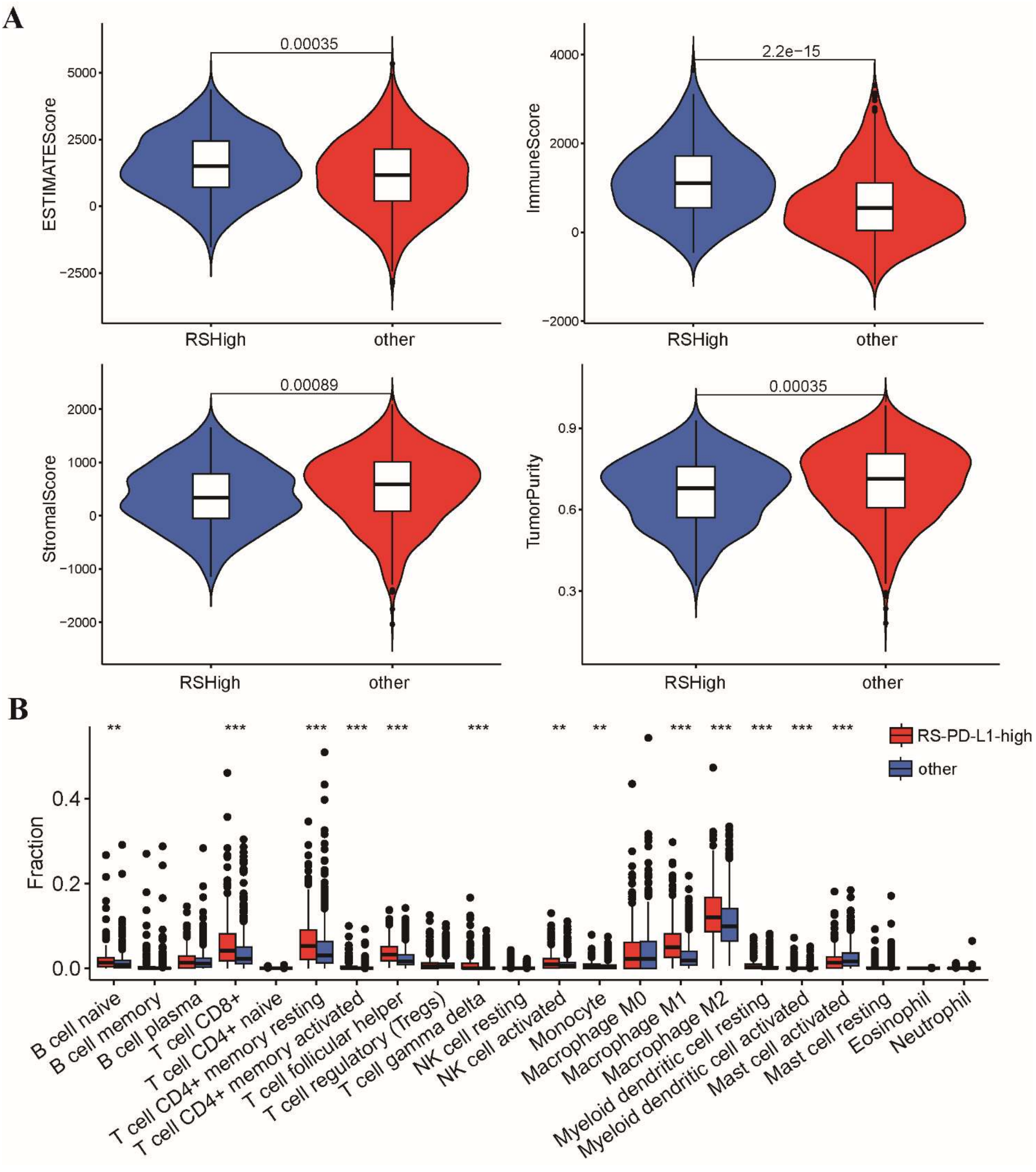

FIGURE S4 (A) The levels of infiltration for estimate scores, stromal scores, immune scores, and tumor purity between the RS-PD-L1-high group and all other group. **(B)** The proportion of 22 immune-infiltrating cells between the RS-PD-L1-high group and all other group. ** p<0.01. *** p<0.001.

TABLE S1 | Sequences of the primer used for qRT-PCR

mRNA	Forward primer	Reverse primer
EMILIN1	CGAGGTTTCAGCCTCTACACAG	CCATCCTCAAGGACACAGCTCA
CYP4Z1	CTGCTTGGCAAAGTACCCTGAG	GTGGTGTAAGGCATCTGGCTCA
GAPDH	GTCTCCTCTGACTTCAACAGCG	ACCACCCTGTTGCTGTAGCCAA

TABLE S2 The coefficients of five variables

Variable	P value
MXRA8	0.010854245
PCOLCE	0.05133559
EMILIN1	0.23810614
NR2F1	0.10013593
CYP4Z1	0.047497493

TABLE S3 Results of PH assumption tests for 15 SRGs in LASSO-Cox regression analysis

Variable	P value
NDN	0.2074
PDGFRB	0.333904
LOXL1	0.17511
COL16A1	0.249346
COL6A1	0.495986
COL1A1	0.37982
OLFML2B	0.832814
MXRA8	0.150928
PCOLCE	0.546227
COL6A2	0.897604
AEBP1	0.555735
CLEC11A	0.056921
EMILIN1	0.792897

NR2F1	0.354867
CYP4Z1	0.979517
GLOBAL	0.09258

TABLE S4 Linear regression algorithm of 10-genes RSI signature

RSI gene	Coefficient
AR	-0.0098009
JUN	0.0128283
STAT1	0.0254552
PRKCB	-0.0017589
RELA	-0.0038171
ABL1	0.1070213
SUMO1	-0.0002509
CDK1	-0.0092431
HDAC1	-0.0204469
IRF1	-0.0441683

TABLE S5 Association between radiosensitivity group and clinicopathological characteristics.

Covariates	Type	Total	RR	RS	P value
Age	<=50	293(31.85%)	142(31.42%)	151(32.26%)	0.8371
	>50	627(68.15%)	310(68.58%)	317(67.74%)	
Radiation	YES	528(57.39%)	264(58.41%)	264(56.41%)	0.5853
	NO	392(42.61%)	188(41.59%)	204(43.59%)	
Menopause	Indeterminate	33(3.59%)	8(1.77%)	25(5.34%)	0.0387
	Peri	35(3.8%)	18(3.98%)	17(3.63%)	
	Post	587(63.8%)	291(64.38%)	296(63.25%)	
	Pre	210(22.83%)	103(22.79%)	107(22.86%)	
	unknow	55(5.98%)	32(7.08%)	23(4.91%)	
Clinical stage	Stage I	157(17.07%)	76(16.81%)	81(17.31%)	0.183
	Stage II	521(56.63%)	243(53.76%)	278(59.4%)	
	Stage III	207(22.5%)	115(25.44%)	92(19.66%)	
	Stage IV	15(1.63%)	10(2.21%)	5(1.07%)	
	unknow	20(2.18%)	8(1.77%)	12(2.56%)	
M stage	M0	763(82.93%)	362(80.09%)	401(85.68%)	0.078
	M1	17(1.85%)	10(2.21%)	7(1.5%)	

	MX	140(15.22%)	80(17.7%)	60(12.82%)	
N stage	N0	435(47.28%)	197(43.58%)	238(50.85%)	0.117
	N1	307(33.37%)	156(34.51%)	151(32.26%)	
	N2	101(10.98%)	54(11.95%)	47(10.04%)	
	N3	62(6.74%)	38(8.41%)	24(5.13%)	
	NX	15(1.63%)	7(1.55%)	8(1.71%)	
T stage	T1	246(26.74%)	131(28.98%)	115(24.57%)	0.0816
	T2	522(56.74%)	240(53.1%)	282(60.26%)	
	T3	121(13.15%)	66(14.6%)	55(11.75%)	
	T4	28(3.04%)	15(3.32%)	13(2.78%)	
	TX	3(0.33%)	0(0%)	3(0.64%)	
